# Megaloblastic Anemia with Ring Sideroblasts is not Always Myelodysplastic Syndrome

**DOI:** 10.4274/tjh.2016.0090

**Published:** 2016-12-01

**Authors:** Neha Chopra Narang, Mrinalini Kotru, Kavana Rao, Meera Sikka

**Affiliations:** 1 University College of Medical Sciences, Department of Pathology, Delhi, India; 2 University College of Medical Sciences, Department of Hematopathology, Delhi, India

**Keywords:** Ring sideroblasts, Megaloblastic anemia, myelodysplastic syndrome

## To the Editor,

Ring sideroblasts are morphological hallmarks of hereditary and acquired sideroblastic anemias [1]. The International Working Group on Morphology of Myelodysplastic syndrome (MDS) defined ring sideroblasts as erythroblasts in which a minimum of five siderotic granules cover at least one-third of the circumference of the nucleus.

**Figure 1:**
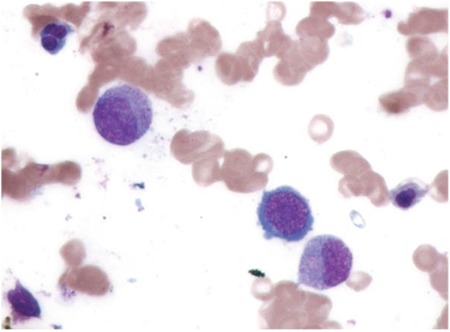
Bone marrow aspiration: megaloblastic maturation with dyserythropoiesis and giant myelocyte (1000^x^).

**Figure 2:**
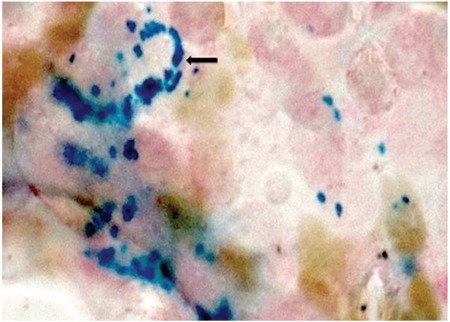
Ring sideroblasts; Perl’s stain on bone marrow aspirate (1000^x^).

We present the case of an 18-year-old female who had low-grade fever, jaundice, nausea, vomiting, and shortness of breath for 25 days. The patient was not an alcoholic and not on any drugs. On examination she appeared pale and icteric; however, no hepatosplenomegaly was noted. A complete blood count (CBC) and bone marrow examination were performed. The CBC revealed Hb: 75 g/L, PCV: 0.232%, RBC: 2.15x10^12^/L, MCV: 108 fL, MCH: 34.8 pg, MCHC: 32.2 g/dL, total leukocyte count: 2.6x10^9^/L, platelet count: 87x10^9^/L, reticulocyte count: 0.8%, and differential leukocyte count: N74 L26. A peripheral smear revealed pancytopenia with dimorphic anemia. No coarse basophilic stippling was noted (as seen in lead poisoning). Bone marrow aspirate was particulate and hypercellular for age with erythroid hyperplasia, showing megaloblastic maturation and dyserythropoiesis (Figure 1). Giant myeloid forms were seen. Megakaryocytes appeared adequate and were normal in morphology. Bone marrow iron was increased (grade 3) and showed 6%-7% ring sideroblasts (Figure 2). A final diagnosis of megaloblastic anemia with ring sideroblasts was made after excluding various other causes of the same symptoms. The patient was put on a therapeutic trial of hematinics (vitamin B12, folic acid, and pyridoxine) and showed improvement. After therapy, a CBC revealed Hb: 122 g/L, PCV: 0.432%, RBC: 4.15x10^12^/L, MCV: 85 fL, MCH: 30.8 pg, MCHC: 31.2 g/dL, total leukocyte count: 5.6x10^9^/L, and platelet count: 177x109/L. However, a repeat bone marrow examination could not be performed as the patient did not comply.

Ring sideroblasts are found exclusively in pathological conditions and should not be confused with ferritin sideroblasts, which are present in normal bone marrow. The latter are normal erythroblasts that, upon Prussian blue staining, show a few blue granules scattered in the cytoplasm, representing endosomes filled with excess iron not utilized for heme synthesis (siderosomes). While the iron of ferritin sideroblasts is stored in cytosolic ferritin, whose subunits are encoded by the FTH1 and FTL genes, the iron of ring sideroblasts is stored in mitochondrial ferritin, encoded by the FTMT gene [2]. There are two forms of sideroblastic anemia: congenital sideroblastic anemia and acquired sideroblastic anemia. Most acquired sideroblastic anemia cases were included within MDS. Acquired sideroblastic anemia in MDS is categorized either as refractory cytopenia with multilineage dysplasia or refractory anemia with ring sideroblasts, depending on the level of dysplasia [3]. Causes of acquired reversible sideroblastic anemia include alcohol use (most common), pyridoxine deficiency, lead poisoning, copper deficiency, excess zinc that can indirectly cause sideroblastic anemia by decreasing absorption and increasing excretion of copper, and antimicrobials like isoniazid, chloramphenicol, linezolid, and cycloserine [1,4].

Impaired heme synthesis in sideroblastic anemias is associated with abnormal vitamin B6 metabolism at the level of the mitochondrion. Megaloblastic anemia due to folic acid deficiency and ringed sideroblastic anemia have been reported in alcohol abusers [1,5,6,7]. Vitamin B6 deficiency is associated with the development of ring sideroblasts in these patients. Patients with megaloblastic anemia showing the presence of ring sideroblasts should therefore be supplemented with pyridoxine in addition to vitamin B12 and folic acid [8]. The presence of ring sideroblasts does not always point towards impending MDS.

The development of ring sideroblasts in the above case was related to an absolute or relative deficiency of pyridoxine associated with vitamin B12 and folate deficiency.
